# Development of a growth-coupled selection platform for directed evolution of heme biosynthetic enzymes in *Corynebacterium glutamicum*

**DOI:** 10.3389/fbioe.2023.1236118

**Published:** 2023-08-15

**Authors:** Yingyu Zhou, Jiuzhou Chen, Wei Pu, Ningyun Cai, Bin Che, Jinxing Yang, Mengmeng Wang, Shasha Zhong, Xingtao Zuo, Depei Wang, Yu Wang, Ping Zheng, Jibin Sun

**Affiliations:** ^1^ College of Biotechnology, Tianjin University of Science and Technology, Tianjin, China; ^2^ Key Laboratory of Engineering Biology for Low-Carbon Manufacturing, Tianjin Institute of Industrial Biotechnology, Chinese Academy of Sciences, Tianjin, China; ^3^ National Technology Innovation Center of Synthetic Biology, Tianjin, China; ^4^ School of Biology and Biological Engineering, South China University of Technology, Guangzhou, China

**Keywords:** heme, growth-coupled, coproporphyrin ferrochelatase, directed evolution, electron transport system, detoxification

## Abstract

Heme is an important tetrapyrrole compound, and has been widely applied in food and medicine industries. Although microbial production of heme has been developed with metabolic engineering strategies during the past 20 years, the production levels are relatively low due to the multistep enzymatic processes and complicated regulatory mechanisms of microbes. Previous studies mainly adopted the strategies of strengthening precursor supply and product transportation to engineer microbes for improving heme biosynthesis. Few studies focused on the engineering and screening of efficient enzymes involved in heme biosynthesis. Herein, a growth-coupled, high-throughput selection platform based on the detoxification of Zinc-protoporphyrin IX (an analogue of heme) was developed and applied to directed evolution of coproporphyrin ferrochelatase, catalyzing the insertion of metal ions into porphyrin ring to generate heme or other tetrapyrrole compounds. A mutant with 3.03-fold increase in *k*_cat_/*K*_M_ was selected. Finally, growth-coupled directed evolution of another three key enzymes involved in heme biosynthesis was tested by using this selection platform. The growth-coupled selection platform developed here can be a simple and effective strategy for directed evolution of the enzymes involved in the biosynthesis of heme or other tetrapyrrole compounds.

## 1 Introduction

Heme, an important tetrapyrrole compound and a versatile prosthetic group in electron transport system (ETS), has been widely applied in daily life, medicine, health and future food (Hansson and Hederstedt, 1994; Chau et al., 2011; Phillips, 2019). Recently, bioproduction of heme by engineered microbes has attracted intensive attention due to its simple and environmentally friendly production processes (Zhao et al., 2018). *Corynebacterium glutamicum* (Ko et al., 2021), *Escherichia coli* (Zhao et al., 2018), and *Saccharomyces cerevisiae* (Liu et al., 2014) are the commonly used host for bioproduction of heme. 5-Aminolevulinic acid (5-ALA) is the precursor of heme, and can be synthesized from succinyl-CoA and glycine via the C4 pathway, or from l-glutamate via the C5 pathway (Dailey et al., 2017). Subsequently, 5-ALA is converted to heme via seven enzymatic reactions (Layer, 2021). In this multistep enzymatic processes, the heme biosynthesis is usually subjected to strict regulation, such as repression of gene expression by transcriptional regulators and feedback inhibition of enzymes by the intermediates and end-product (Wang et al., 1997; Zhang et al., 2015; Krüger et al., 2022). For example, HrrA (regulated by transcriptional regulator DtxR in *C. glutamicum*) was found to repress almost all genes involved in heme biosynthesis (Frunzke et al., 2011). The enzyme activity of hydroxymethylbilane synthase (encoded by the *hmbS*) from *Vibrio cholerae* decreased by approximately 15% with heme binding (Uchida et al., 2018). Previous studies mainly focused on enhancing precursor supply and heme biosynthesis pathways by overexpressing the key genes with multi-copy plasmids or stronger promoters to improve heme production (Zhao et al., 2018; Ko et al., 2021). Although these strategies have promoted heme biosynthesis, the intrinsic low activity and feedback inhibition of key biosynthetic enzymes are still limiting factors for heme overproduction. Moreover, the catalytic and regulatory mechanisms of enzymes involved in heme biosynthesis are complex, unclear, and highly diverse among different organisms (Zamarreño Beas et al., 2022), which limited the engineering of these enzymes by rational design.

Directed evolution coupled with high-throughput screening is an effective strategy for engineering enzymes, which does not depend on the structural and functional information of target enzymes (Xiao et al., 2015). Ferrochelatase catalyzes the reaction inserting metal ions into porphyrin ring to generate heme or other tetrapyrrole compounds (Dailey et al., 2000). Its substrate protoporphyrin IX (PPIX) exhibits fluorescence output at 650 nm, while its product with integrated zinc shows significantly decreased fluorescence output. Taking advantage of the fluorescence change, directed evolution of the ferrochelatase from *Bacillus subtilis* was conducted by using fluorescence activated cell sorting (FACS)-based high-throughput screening strategy, five mutants with 1.15- to 2.95-fold higher *k*_cat_/*K*_M_ compared to the wild-type enzyme were obtained and characterized *in vitro* (Kwon et al., 2004). Recently, a heme biosensor based on the heme-responsive transcriptional regulator HrtR and its target operator HrtO was developed (Zhang et al., 2020). By using the tetracycline efflux protein TcR as a reporter, the intracellular heme concentration was coupled with the cellular resistance to tetracycline, which decided the cell growth rate under appropriate tetracycline stress. This biosensing system was also successfully applied to directed evolution of ferrochelatase from *B. subtilis*, and 14 mutants with increased cellular resistance to tetracycline as well as a high-activity variant with 2.04-fold higher heme production were obtained from a saturation mutagenesis library (Zhang et al., 2023).

Establishing a correlation between cell growth and heme production, which allows growth-coupled *in vivo* selection, provided an effectively high-throughput screening model for the evolution of heme biosynthetic enzymes. Except for the biosensor-based growth-coupled selection strategy, engineering synthetic auxotrophs and utilization or detoxification of substrates were also the common strategies for growth-coupled selection (Zhang et al., 2021; Chen et al., 2022; Nielsen et al., 2023). Considering that heme plays an important role in electron transfer, aerobic respiration, and oxygen metabolism as a prosthetic group of cytochromes and catalases (Ajioka et al., 2006; Layer et al., 2010), a quantitative relationship between cell growth rate and activity of heme biosynthetic enzymes can be established independent of the transcriptional regulator-based biosensor and complex genetic circuits. In this study, a growth-coupled selection platform for directed evolution of heme biosynthetic enzymes was developed based on the detoxification of non-iron metalloporphyrins (MPs) in *C. glutamicum*. Firstly, we tested the effects of Zinc-protoporphyrin IX (ZnPPIX) on the growth of *C. glutamicum*. Then the sensitivity of cell growth was further improved by enhancing the ZnPPIX uptake and the role of this selection system was confirmed by adding 5-ALA, the precursor of heme. Finally, this high-throughput selection system was successfully applied to directed evolution and high-throughput screening four key enzymes involved in heme biosynthesis in *C. glutamicum*, and several superior coproporphyrin ferrochelatase (CpfC) mutants with improved catalytic activity were obtained. The growth-coupled selection platform developed in this study may also be a useful strategy for directed evolution of other enzymes involved in heme or other tetrapyrrole compounds biosynthesis, and providing more efficient catalytic elements for facilitating the heme or other tetrapyrrole compounds overproduction.

## 2 Materials and methods

### 2.1 Bacterial strains and cultivation conditions

The bacterial strains, plasmids, and primers used in this study are listed in Supplementary Table S2, S3. *E. coli Trans*1-T1 (TransGen Biotech Co. Ltd., Beijing, China) was used as the host for plasmid construction and was cultivated at 37°C using Luria-Bertani (LB) medium. *E. coli* BL21 (TransGen Biotech Co. Ltd., Beijing, China) was used as the host for protein-induced expression and was cultivated at 16°C using LB medium. *C. glutamicum* ATCC 13032 was cultivated at 30°C in TSB medium (Liu et al., 2022) or CGXII minimal medium supplemented with appropriate glucose (Wang et al., 2018). ZnPPIX (16 μg/mL), sodium gluconate (10 g/L), chloramphenicol (Cm, 5 μg/mL), kanamycin (Km, 25 μg/mL), and isopropyl-β-d-thiogalactopyranoside (IPTG, 0.1 mM) were used for *C. glutamicum* as required, respectively. Cm (20 μg/mL), Km (50 μg/mL), ampicillin (Amp, 100 μg/mL), and IPTG (0.5 mM) were used for *E. coli* as required, respectively.

### 2.2 Generation of CpfC random mutation library

To obtain the random mutation PCR fragment of *cpfC*, a standard error-prone PCR protocol was employed by using *Taq* polymerase with varied concentrations of MnCl_2_ (0.05 mM, 0.1 mM, 0.2 mM, 0.3 mM, 0.4 mM, and 0.5 mM). The error-prone PCR fragments were ligated with the linearized pEC-P_*gntK*_ (substituting the IPTG-inducible promoter P_*trc*_ of the plasmid pEC-XK99E to a gluconate-inducible promoter P_*gntK*_). The CpfC mutation library was constructed by recombination, and then transformed into strain *C. glutamicum* ATCC 13032 (pXMJ19-*hmuTUV*) through electroporation.

### 2.3 Growth-coupled selection of CpfC mutant library

To screen the desired CpfC mutants from the library, the transformants were cultivated in TSB medium with adding Cm and Km. After about 24 h of cultivation, the cultivated cells were washed twice with CGXII minimal medium, then used to inoculate 800 μL CGXII minimal medium supplemented with 40 g/L glucose, 0.1 mM IPTG, 10 g/L sodium gluconate, 16 μg/mL ZnPPIX, Cm, and Km in 24-well culture plates with an initial OD_600nm_ (optical density at 600 nm) of 0.1. The cultures were incubated at 30°C with shaking at 800 rpm. Subsequently, the cells were properly diluted and spread on CGXII agar plate with adding 40 g/L glucose, 0.1 mM IPTG, 10 g/L sodium gluconate, 16 μg/mL ZnPPIX, Cm, and Km. Ten big colonies were randomly picked and used for secondary verification under the same culture conditions described above. Finally, three strains with improved growth were further investigated after two rounds of growth tests in 24-well culture plates.

### 2.4 Enzyme activity assays

Determination of the enzyme activity of CpfC was referred to the methods established by Porra, et al. (Porra and Lascelles, 1968). The reactions were performed in bath incubator at 30°C with 200 μL standard reaction mixture containing 66 mM Tris-HCl (pH 8.0), 3.3% Tween 20, 5 mM glutathione, 100 μM 2-mercaptoethanol, 100 μM ferrous ammonium sulfate, 50 μM substrate (coproporphyrin III, CPIII), appropriate purified enzyme extracts for 10 min, and stopped by adding same volume trichloroacetic acid. Then, the sample was centrifuged (12,000 × g, 10 min, 4°C), and the 100 μL supernatant was transferred to a clean tube, which contained 900 μL ddH_2_O. Subsequently, 200 μL of the supernatant was added to a 96-well microtiter plate and the specific activity was determined using a microplate reader (SpectraMax M5, Molecular Devices, λ excitation = 395 nm, λ emission = 611 nm). *K*_M_ values were determined by nonlinear regression analysis using the Michaelis-Menten kinetic model with various substrate concentrations. The standard samples containing different concentrations (0 μM, 20 μM, 40 μM, 60 μM, 80 μM, 100 μM, 120 μM, 140 μM) of CPIII were measured using the method described above. A standard curve was drawn and the CPIII concentration was determined from the standard curve. One unit (U) of CpfC activity was defined as the amount of enzyme that converted 1 μM of CPIII into coproheme per minute. The method developed by Hansson, et al. (Hansson et al., 2006), which employed PPIX as the substrate, was used to measure the enzyme activity.

## 3 Results

### 3.1 Design and construction of a growth-coupled selection system based on the detoxification of MPs

**FIGURE 1:**
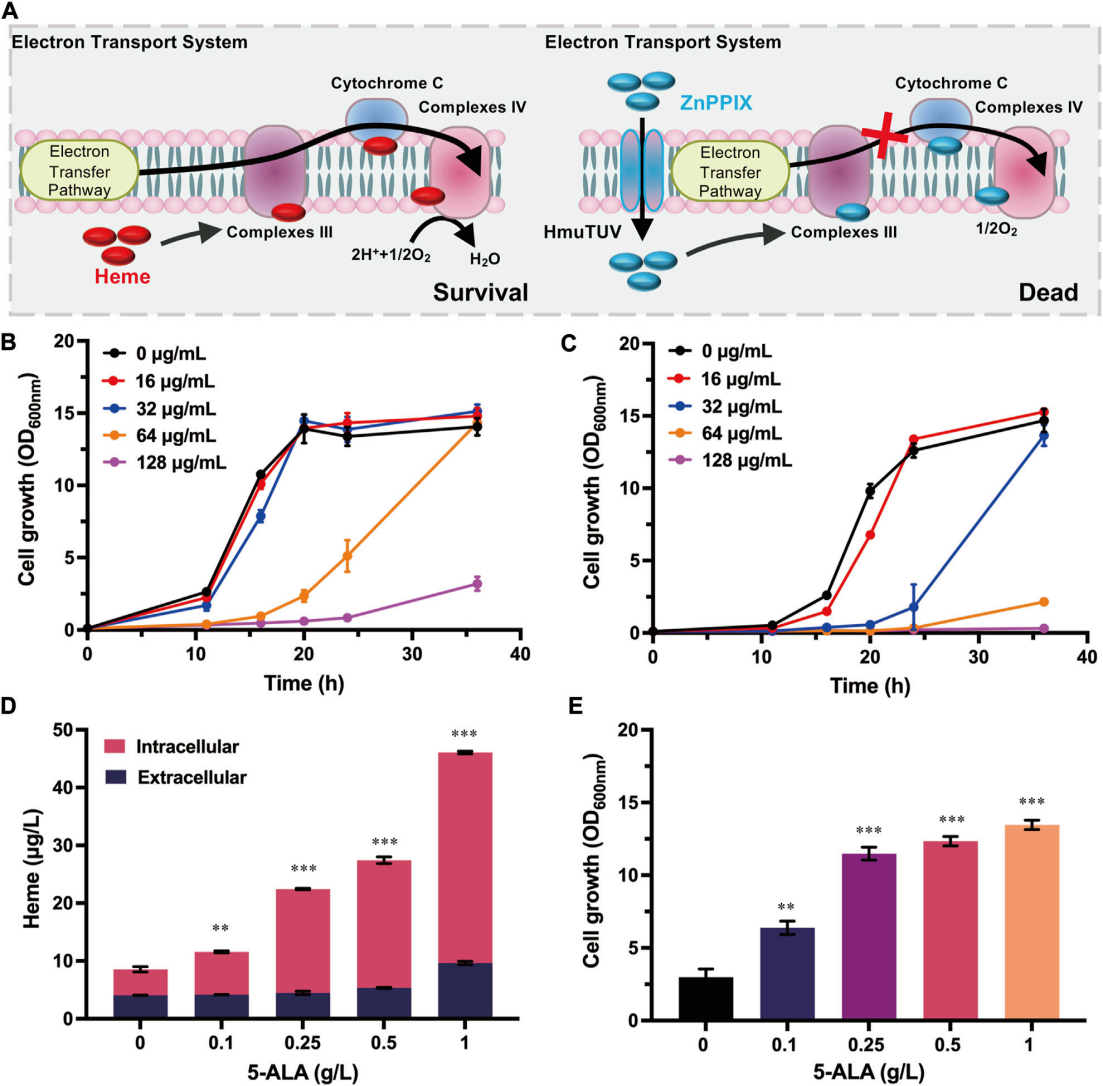
Design and characterization of the growth-coupled selection platform in *C. glutamicum*. **(A)** Schematics of the growth-coupled selection system based on the detoxification of ZnPPIX. In the normal ETS, cytochrome that binds heme was the carrier for electron transport. As the analogues of heme, ZnPPIX could bind cytochrome instead with heme. When the concentration of ZnPPIX was elevated, the electron transfer was inhibited and the cell growth was retarded. The inhibition of ZnPPIX could be reversed by excessive heme produced. Heme is highlighted in red, ZnPPIX is highlighted in blue. **(B)** Cell growth of *C. glutamicum* ATCC 13032 with addition of different concentrations of ZnPPIX. **(C)** Cell growth of the *hmuTUV* overexpressing strain with addition of different concentrations of ZnPPIX. **(D)** The extracellular and intracellular heme concentrations of the *hmuTUV* overexpressing strain with adding different concentrations of 5-ALA and 32 μg/mL ZnPPIX at 24 h. Detailed assay conditions are described in the supplementary materials and methods section. **(E)** Cell growth of the *hmuTUV* overexpressing strain with supplementing different concentrations of 5-ALA and 32 μg/mL ZnPPIX at 24 h. Data are presented as mean values +/− SD (n = 3 independent experiments). 0.001 < **p < 0.01, ***p < 0.001, student’s two-tailed *t*-test.

Based on the special property of heme, a series of heme analogues have been explored and used as antibacterial agents. MPs, a kind of heme analogues with a metal ion other than iron, possess a strong antibacterial activity against several pathogenic bacteria (Stojiljkovic et al., 1999). MPs is likely to utilize the heme-uptake routes to enter cells, then it could competitively bind heme-containing enzymes, generally resulting in the inhibition of cellular aerobic respiration by targeting cytochromes (Hijazi et al., 2017). Meanwhile, the inhibition could be reversed by excessive heme exogenously added or produced by cells (Stojiljkovic et al., 1999), indicating that the intracellular heme could compete with MPs to restore the ETS and the cell growth. Therefore, MPs was used to serve as a select pressure to develop a growth-coupled selection strategy based on the restoration of ETS for directed evolution of enzymes involved in heme biosynthesis (Figure 1A).

Since the effect of MPs on *C. glutamicum* ATCC 13032 remained unclear, the cell growth of wild-type strain under different concentrations of ZnPPIX was firstly investigated. Cell growth of *C. glutamicum* was not significantly inhibited by 32 μg/mL ZnPPIX. When adding with 64 μg/mL ZnPPIX, the cell growth of *C. glutamicum* began to be inhibited, and the biomass at 24 h only reached 38.2% of that adding 0 μg/mL ZnPPIX. With the increase of ZnPPIX concentration, the cell growth was further retarded (Figure 1B). Although ZnPPIX exhibited toxicity to cells, growth inhibition only occurred at relatively high concentrations. Considering that the uptake of porphyrins required specific transport systems, enhancing the endogenous heme-uptake system by overexpressing *hmuTUV* may improve the sensitivity of cells to ZnPPIX. As expected, the sensitivity of the *hmuTUV* overexpressing strain to ZnPPIX was elevated compared to the control strain (Figure 1C). Cell growth was significantly retarded in the presence of 32 μg/mL ZnPPIX, reaching only 12.8% of the biomass observed in the control strain at 32 μg/mL ZnPPIX after 24 h (Figure 1C). These results manifest that the sensitivity of *C. glutamicum* to MPs is also depended on the heme-uptake system, and overexpression of HmuTUV drastically decreased the working concentration of the ZnPPIX.

The inhibition of ZnPPIX could be reversed by excessive heme exogenously added or produced by cells (Stojiljkovic et al., 1999). 5-ALA is a common precursor of porphyrin and heme in all living organisms (Zhu et al., 2019), and is beneficial for heme production. To verify the feasibility of the growth-coupled selection platform, different concentrations of 5-ALA were added to test the cell growth of *C. glutamicum* in the presence of ZnPPIX. The results showed that adding 5-ALA promoted heme biosynthesis. With the increase of 5-ALA addition, the concentration of intracellular and extracellular heme gradually increased (Figure 1D). The resistance of cells to ZnPPIX was also enhanced, leading to improved cell growth under the stress of 32 μg/mL ZnPPIX (Figure 1E). The improved cell growth may be attributed to the detoxification of ZnPPIX by heme, which competes with ZnPPIX to bind heme-containing enzymes in the ETS. When the ZnPPIX-based selection system was used for directed evolution of the key enzymes involved in heme biosynthesis, mutants contributed to heme biosynthesis would be enriched with the selection platform. Therefore, a growth-coupled selection platform based on the detoxification of ZnPPIX was successfully developed in *C. glutamicum* for the first time, which may be useful for directed evolution of enzymes involved in heme biosynthesis.

### 3.2 Overexpression of heme biosynthetic enzymes compensates for cell growth with ZnPPIX stress

**FIGURE 2:**
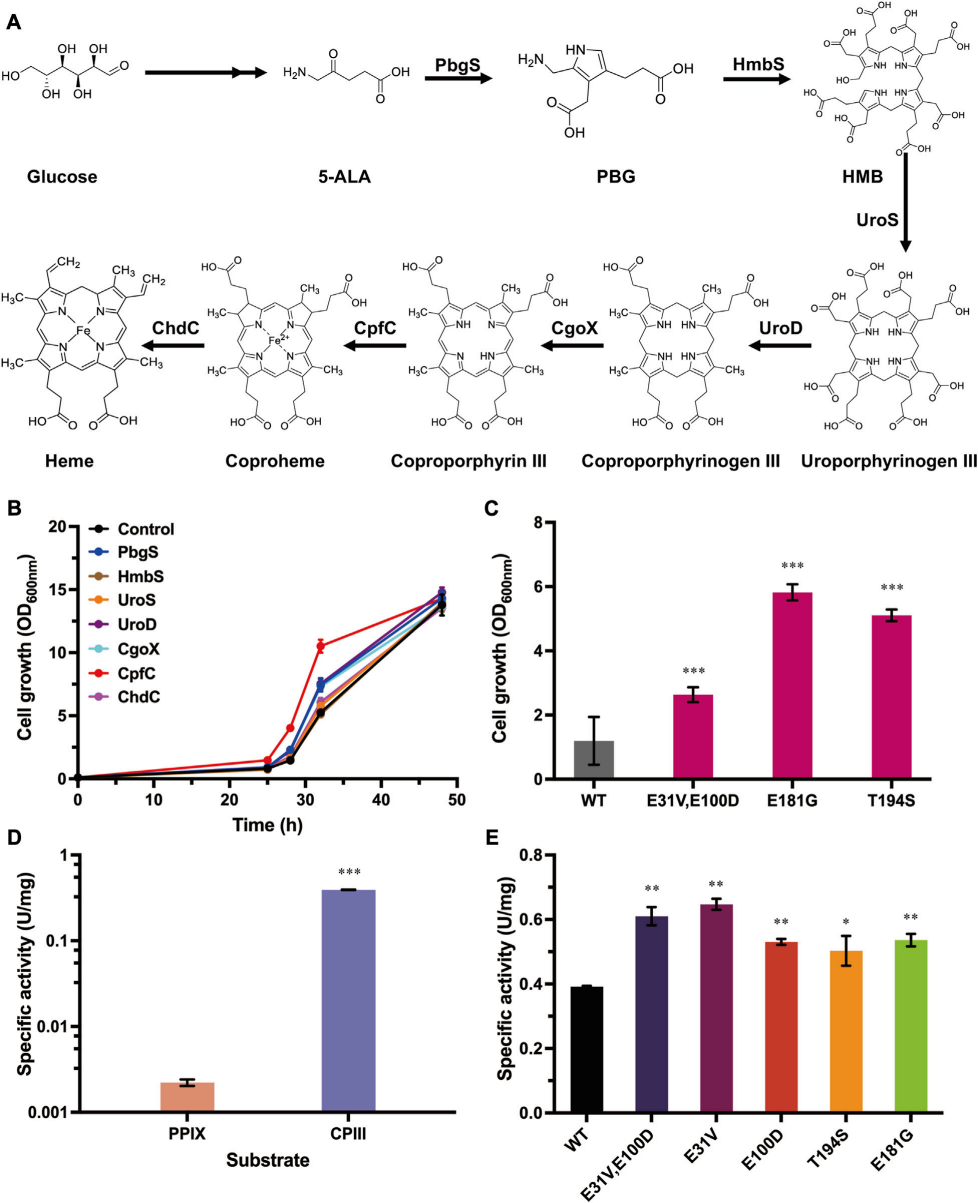
Overexpression of heme biosynthetic enzymes compensating for cell growth and directed evolution of CpfC with the growth-coupled selection system. **(A)** Biosynthesis pathway of heme in *C. glutamicum*. Heme is synthesized from 5-ALA via seven enzymatic reactions. PbgS (Porphobilinogen synthase), HmbS (Hydroxymethylbilane synthase), UroS (Uroporphyrinogen synthase), UroD (Uroporphyrinogen decarboxylase), CgoX (Coproporphyrinogen oxidase), CpfC (Coproporphyrin ferrochelatase), and ChdC (Coproheme decarboxylase). **(B)** Cell growth of the strains overexpressing genes involved in heme biosynthesis. The strain harboring plasmids pXMJ19-*hmuTUV* and pEC-P_*gntK*_ was used as the control. These strains were cultivated in CGXII medium with adding 40 g/L glucose, 16 μg/mL ZnPPIX, 10 g/L sodium gluconate, 0.1 mM IPTG, 25 μg/mL Km, and 5 μg/mL Cm. **(C)** Cell growth of the reconstructed strains overexpressing *cpfC* mutants. The strain harboring plasmids pXMJ19-*hmuTUV* and pEC-P_*gntK*_-*cpfC* ^WT^ was used as the control. Cultivation conditions were same as the above. **(D)** The specific activities of CpfC^WT^ were measured with different substrates. **(E)** The specific activities of CpfC mutants. Detailed assay conditions are described in the methods section. Data are presented as mean values +/− SD (n = 3 independent experiments). 0.01 < *p < 0.05, 0.001 < **p < 0.01, ***p < 0.001, student’s two-tailed *t*-test.

To select the enzymes that can be used for evolution, all seven enzymes (PbgS, HmbS, UroS, UroD, CgoX, CpfC, and ChdC) involved in heme biosynthesis form 5-ALA (Figure 2A) were systematically investigated for their influence on cell growth with the above-established growth-coupled selection system. To eliminate the mutual interference between the uptake system of ZnPPIX and these enzymes, these genes (*pbgS*, *hmbS*, *uroS*, *uroD*, *cgoX*, *cpfC* and *chdC*) were overexpressed in another plasmid pEC-P_*gntK*_ (substituting the IPTG-inducible promoter P_*trc*_ of the plasmid pEC-XK99E to a gluconate-inducible promoter P_*gntK*_). Considering that adding two antibiotics to maintain the two plasmids (pXMJ19*-hmuTUV* and pEC-P_*gntK*_ derivates) may also influence the cell growth in the medium with adding ZnPPIX, the engineered strain harboring plasmids pXMJ19-*hmuTUV* and pEC-P_*gntK*_ was retested under different concentrations of ZnPPIX. The results showed that this strain was more sensitive to ZnPPIX than the strain only harboring pXMJ19-*hmuTUV* (Supplementary Figure S1). Addition of 16 μg/mL of ZnPPIX produced a significant inhibitory effect. Therefore, 16 μg/mL ZnPPIX was used in the following experiments. Then the cell growth of strains harboring the plasmids pXMJ19-*hmuTUV* and pEC-P_*gntK*_ overexpressing different genes involved in heme biosynthesis were tested. Overexpression of *cpfC*, *pbgS*, *uroD*, and *cgoX* genes showed improved cell growth compared with the control strain harboring plasmids pXMJ19-*hmuTUV* and pEC-P_*gntK*_. The strain overexpressing *cpfC* showed the highest growth rate amongst all the tested strains. The biomass was 1.81-fold higher than that of the control strain at 24 h (Figure 2B), which was consistent with the previous study that ferrochelatase was a rate-limiting enzyme for heme biosynthesis in *E. coli* (Zhao et al., 2018). These results indicate that the enhancement of PbgS, UroD, CgoX and CpfC expression may promote heme biosynthesis, which contributed to improving the cell growth with ZnPPIX stress.

### 3.3 Directed evolution of CpfC with the growth-coupled selection system

Among all the tested enzymes involved in heme biosynthesis, overexpressing *cpfC* showed the highest growth rate, so it was selected to verify the availability of the growth-coupled selection system. To evolve CpfC, a random mutation library of CpfC was constructed by error-prone PCR. The plasmid library with a size of 10^6^ CpfC mutants was then transformed into *C. glutamicum* harboring plasmid pXMJ19-*hmuTUV*, and the cells were subjected to the established growth-coupled selection in CGXⅡ minimal medium with adding 16 μg/mL ZnPPIX. After 24 h cultivation, the biomass of the culture containing the CpfC mutants was 2.83-fold higher than that of the control strain overexpressing the wild-type CpfC (CpfC^WT^) (Supplementary Figure S2), suggesting that the positive mutants contributing to improved cell growth may be enriched in the mixed culture. Then, the culture was screened on CGXⅡ agar plates with 16 μg/mL ZnPPIX. As a control, cells harboring plasmids pXMJ19-*hmuTUV* and pEC-P_*gntK*_-*cpfC* were spread on the CGXII agar plate supplemented with ZnPPIX, and no clones grew (Supplementary Figure S3). However, for the CpfC mutant library, clones with different sizes were obtained. Subsequently, ten clones with relatively large sizes were picked to further test the tolerance to ZnPPIX in 24-well plates. All of the ten clones showed improved growth under the test conditions. Sequences of the ten variants were characterized and three types of mutations were identified. To remove the possible mutations in the plasmid backbone or the chromosome that were randomly introduced during the evolution process, the expressing plasmids were reconstructed based on the sequencing data and transformed into strain *C. glutamicum* harboring plasmid pXMJ19-*hmuTUV* for test. The strains harboring the reconstructed CpfC mutants showed 2.20- to 4.87- fold higher biomass than the strain harboring CpfC^WT^ after 24 h cultivation (Figure 2C). These results indicate that the improved tolerance of the evolved strains to ZnPPIX did result from the expression of CpfC mutants.

### 3.4 Enzyme activity assay of the CpfC mutants

To verify whether the enhanced ZnPPIX tolerance was caused by the improved activities of the CpfC mutants, the CpfC^WT^ from *C. glutamicum* and mutants were heterologously overexpressed in *E. coli*, purified, and used for the characterization of the enzymatic properties. Previous studies have demonstrated that Gram-positive bacteria utilize the coproporphyrin-dependent (CPD) pathway to synthesize heme (Dailey et al., 2015; Hobbs et al., 2017; Gabler et al., 2022), revealing that the actual substrate for ferrochelatases from these bacteria is CPIII instead of PPIX. *C. glutamicum* is a Gram-positive bacterium, previous study confirmed the possibility of CPD pathway in *C. glutamicum* by *in vitro* analysis with different substrates (Seok et al., 2019). However, the enzymatic properties of CpfC in *C. glutamicum* have not yet been characterized. A fluorescence detection method was firstly constructed based on the fact that CPIII in solution at specific wavelengths is fluorescent, whereas coproheme is not (Porra and Lascelles, 1968). A spectrofluorimetric scanning was conducted for the CPIII and coproheme (dissolved in 0.1 M NaOH), respectively. The CPIII exhibited an obvious fluorescence at λ excitation = 395 nm and λ emission = 611 nm, while coproheme did not (Supplementary Figure S4). There was a linear correlation between CPIII and the fluorescence (Supplementary Figure S5). Therefore, the activity of CpfC and its mutants can be determined by monitoring the changes in concentration of substrate CPIII. Based on this method, the specific activity of CpfC^WT^ from *C. glutamicum* was 0.39 U/mg using the CPIII as substrate (Figure 2D), while the specific activity of CpfC^WT^ with the PPIX as substrate was only 2.22 × 10^−3^ U/mg (Figure 2D), which was two orders of magnitude lower than the former. These results indicate that CPIII is the native substrate for heme biosynthesis in *C. glutamicum*. The *K*_M_ value of CpfC was 15.17 × 10^−3^ mM (Table 1; Supplementary Figure S6), which was similar to previously reported CpfCs from other Gram-positive bacteria *Staphylococcus aureus* and *B. subtilis* (Lobo et al., 2015; Hobbs et al., 2017). Therefore, the following studies all utilized the CPIII as the substrate to characterize the enzymatic properties of CpfC mutants.

**TABLE 1:** Kinetic characterization of CpfC mutants on CPIII.

Mutant	*K*_M_ (× 10^−3^ mM)	*k*_cat_ (S^−1^)	*k*_cat_/*K*_M_ (mM^-1^S^−1^)
WT	15.17 ± 1.84	0.26 ± 0.09	17.43 ± 0.11
E181G	10.04 ± 1.52	0.30 ± 0.04	30.61 ± 4.31
T194S	11.63 ± 1.50	0.32 ± 0.06	27.30 ± 4.88
E31V, E100D	9.78 ± 0.82	0.36 ± 0.04	37.01 ± 4.52
E31V	8.26 ± 0.52	0.43 ± 0.01	52.91 ± 1.40
E100D	9.40 ± 1.50	0.36 ± 0.06	38.10 ± 0.65

Then the activities of the CpfC mutants were measured with the above-established method. The results showed that the specific activities of the mutants CpfC^E31V,E100D^, CpfC^E181G^, and CpfC^T194S^ were 0.61 U/mg, 0.54 U/mg, and 0.50 U/mg, which showed 1.56-fold, 1.37-fold, and 1.29-fold higher than that of the CpfC^WT^, respectively (Figure 2E). The *K*_M_ values of the CpfC mutants were lower than that of the CpfC^WT^ (15.17 × 10^−3^ mM), and the *k*_cat_/*K*_M_ were significantly higher than that of the CpfC^WT^ (17.43 mM^-1^S^−1^) (Table 1). Especially, the *k*_cat_/*K*_M_ of CpfC^E31V,E100D^ (37.01 mM^-1^S^−1^) was 2.12-fold higher than that of the CpfC^WT^ (Table 1). These results indicate that the increased tolerance of CpfC mutants to ZnPPIX is associated with its enhanced enzymatic activities, and demonstrate the practicability of the growth-coupled selection system based on the detoxification of MPs for directed evolution of key enzymes in heme biosynthesis.

To further identify the key amino acid substitution that is essential for improving the activity of the mutant CpfC^E31V,E100D^, single-site mutants CpfC^E31V^ and CpfC^E100D^ were also measured (Figure 2E; Table 1). The specific activities of CpfC^E31V^ and CpfC^E100D^ were 0.65 U/mg and 0.53 U/mg, which were 1.65-fold and 1.35-fold higher than that of the CpfC^WT^, respectively (Figure 2E). Additionally, the *k*_cat_/*K*_M_ of CpfC^E31V^ was up to 52.91 mM^-1^S^−1^ for CPIII, increased by 3.03-fold compared with the CpfC^WT^ (Table 1). These results manifest that the residue E31V is the key residue contributing to the improved enzymatic properties.

### 3.5 Growth-coupled evolution of PbgS, UroD, and CgoX

**FIGURE 3:**
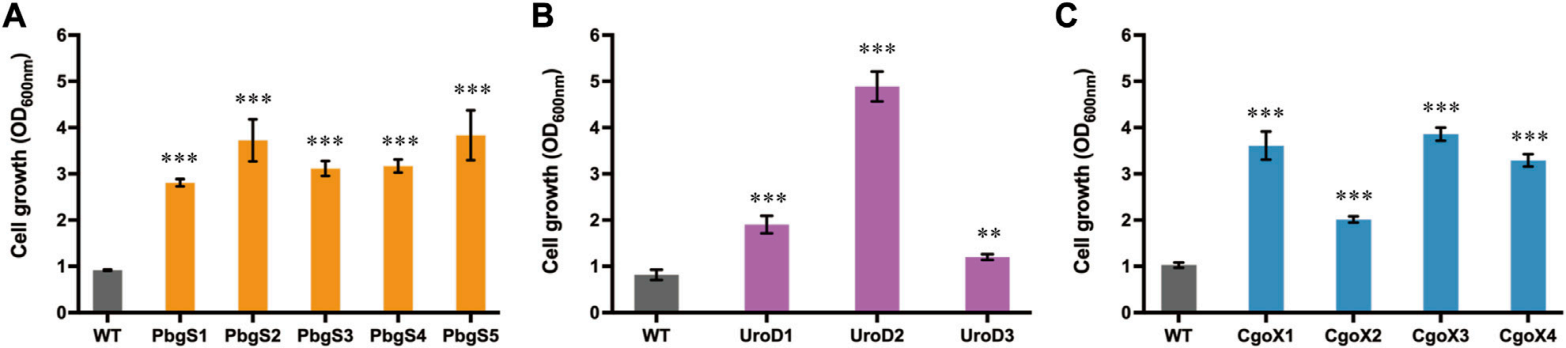
Cell growth of the strains harboring reconstructed mutants of PbgS **(A)**, UroD **(B)**, and CgoX **(C)**. The strains harboring pXMJ19-*hmuTUV* and pEC-P_*gntK*_ overexpressing the wild-type genes were used as the control. These strains were cultivated in CGXII medium with adding 40 g/L glucose, 16 μg/mL ZnPPIX, 10 g/L sodium gluconate, 0.1 mM IPTG, 25 μg/mL Km, and 5 μg/mL Cm. Data are presented as mean values +/− SD (n = 3 independent experiments). 0.001 < **p < 0.01, ***p < 0.001, student’s two-tailed *t*-test.

As shown in Figure 2B, overexpressing *pbgS*, *uroD*, and *cgoX* also exhibited improved growth tendencies, indicating that they are promising candidates for directed evolution using the selection platform to capture the mutants with improved activity. To verify this hypothesis, directed evolutions of these enzymes were performed. Firstly, the random mutation libraries of *pbgS, uroD* and *cgoX* were constructed and subjected to growth-coupled selection, respectively. After 24 h cultivation, the biomasses of strains containing the PbgS, UroD, and CgoX mutation libraries were 7.14-fold, 4.59-fold, and 7.31-fold higher than that of the control strains overexpressing the wild-type genes, respectively (Supplementary Figure S7). Then, the mutants with improved growth were obtained and reconstructed. Finally, the cell growth of these reconstructed strains was tested in CGXII medium with adding 16 μg/mL ZnPPIX. The biomasses of the strains harboring PbgS, UroD, and CgoX mutants were 3.05- to 4.17-fold, 1.46- to 5.96-fold, and 1.95- to 3.75-fold higher than that of the control strains harboring wild-type enzymes at 24 h, respectively (Figure 3; Supplementary Table S3). These results suggest that the growth-coupled selection system based on the detoxification of MPs was an effective strategy for directed evolution of enzymes involved in heme biosynthesis or other tetrapyrrole compounds.

## 4 Discussion

Microbial production of heme has received significant attentions in recent years due to its environmental-friendliness and low cost. To improve heme production, previous studies mainly focused on engineering microorganisms through pathway and transporter engineering. However, few studies focused on the engineering and screening of effective enzymes involved in heme biosynthesis. In this study, based on the physiological function of heme as a cofactor in the ETS, a high-throughput, growth-coupled selection platform was developed and successfully applied to directed evolution four key enzymes for heme biosynthesis in *C. glutamicum*, and efficiently produced several robust mutants with improved activity. This strategy depended on the target product concentration instead of the specific fluorescence of intermediate product (Kwon et al., 2004), enabling it also applicable for screening more enzymes involved in heme or other tetrapyrrole compounds biosynthesis. The growth-coupled selection system developed here was simpler and more effective, and provided an alternative strategy for engineering and screening more key enzymes involved in heme or tetrapyrrole compounds biosynthesis.

Ferrochelatase catalyzes the reaction inserting ferrous iron into a porphyrin ring to generate heme or other tetrapyrrole compounds, and is one of the key rate-limiting enzymes involved in the heme biosynthesis (Pranawidjaja et al., 2015). Therefore, some attempts have been employed to improve its enzyme activity. For example, directed evolution of the ferrochelatase from *B. subtilis* in *E. coli* was conducted by using FACS-based high-throughput screening strategy, the best mutant with 2.95-fold higher *k*_cat_/*K*_M_ compared to the wild-type was obtained (Kwon et al., 2004). However, the enzymatic activity was determined using the PPIX as substrate, which is not the optimal substrate for this enzyme from *B. subtilis*. Dailey and colleagues demonstrated that the actual substrate for ferrochelatase from *B. subtilis* is CPIII instead of PPIX. The noncanonical CPD pathway is responsible for heme biosynthesis in Gram-positive bacteria (Dailey et al., 2015). The CPD pathway for *C. glutamicum* was confirmed by *in vitro* heme biosynthesis from PPIX or CPIII using CpfC and ChdC. When CPIII was added in the crude enzyme extract of strain overexpressing *cpfC* and *chdC*, much higher heme was accumulated than that using PPIX as the substrate (Seok et al., 2019). These results indicate that the CPD pathway is the main heme biosynthetic pathway in *C. glutamicum*. However, the enzymatic properties of CpfC in *C. glutamicum* have not yet been characterized. Herein, a more efficient and sensitive enzymatic assay method was developed based on the fluorescent properties of CPIII. This method was applied to characterize the enzymatic property of CpfC from *C. glutamicum.* Our results showed that the specific activity of CpfC from *C. glutamicum* using CPIII as substrate was two orders of magnitude higher than that of using the PPIX as substrate, which confirmed the CPD pathway in *C. glutamicum* at the enzymatic level for the first time. With the method developed here using the CPIII as substrate, the basic enzymatic properties of CpfC from *C. glutamicum* were characterized, which were similar to the CpfCs from the other Gram-positive bacteria (Dailey et al., 2015; Lobo et al., 2015). Based on the growth-coupled selection system developed in this study, several CpfC mutants with increased catalytic activity were obtained. The key site in these CpfC mutants for improving enzymatic activity was also identified. The best CpfC mutant exhibited 3.03-fold higher *k*_cat_/*K*_M_ than the wild-type enzyme, providing a good catalytic element for heme production.

In conclusion, a simple and effective growth-coupled selection platform for directed evolution of the enzymes involved in heme biosynthesis was developed, and several superior CpfC, PbgS, UroD, and CgoX mutants were successfully acquired, which will serve as better catalytic elements for heme overproduction. However, another three enzymes (HmbS, UroS, ChdC) have not exhibited improved growth tendencies, the selection platform could be optimized by regulating the intensity of gene expression and co-expressing multiple genes to improve the universality. Moreover, the selection system also has the potential to provide new inspirations into broadening our understanding of the catalytic and regulatory mechanisms of enzymes involved in heme or other tetrapyrrole compounds biosynthesis.

## Data Availability

The raw data supporting the conclusion of this article will be made available by the authors, without undue reservation.
