# Supplementary material for: Development of a growth-coupled selection platform for directed evolution of heme biosynthetic enzymes in Corynebacterium glutamicum
Source: Front Bioeng Biotechnol. 2023 Aug 15;11:1236118. doi: 10.3389/fbioe.2023.1236118 (PMC10465345; doi:10.3389/fbioe.2023.1236118)
Supplement: Supplementary file 1 [file DataSheet1.docx]

Supplementary Material

Development of a Growth-Coupled Selection Platform for Directed Evolution of Heme Biosynthetic Enzymes in *Corynebacterium glutamicum*

Yingyu Zhou^1^^,2†^, Jiuzhou Chen^2,3†^, Wei Pu^2,3^, Ningyun Cai^1,2^, Bin Che^2,3^, Jinxing Yang^2,4^, Mengmeng Wang^1,2^, Shasha Zhong^1,2^, Xingtao Zuo^1,2^, Depei Wang^1^, Yu Wang^2,3*^, Ping Zheng^2,3*^ and Jibin Sun^2,3^

^1^College of Biotechnology, Tianjin University of Science and Technology, Tianjin 300457, China

^2^Key Laboratory of Engineering Biology for Low-carbon Manufacturing, Tianjin Institute of Industrial Biotechnology, Chinese Academy of Sciences, Tianjin 300308, China

^3^National Technology Innovation Center of Synthetic Biology, Tianjin 300308, China

^4^School of Biology and Biological Engineering, South China University of Technology, Guangzhou, 510006, China

***Corresponding authors**

Dr. Yu Wang

wang_y@tib.cas.cn

Dr. Ping Zheng

zheng_p@tib.cas.cn

# Supplementary Materials and Methods

## Chemicals and Reagents

All chemicals were purchased from Sangon Biotech (Shanghai, China) and Solarbio Life Science (Beijing, China) unless otherwise specified. ZnPPIX was purchased from Sigma–Aldrich (Shanghai, China). Restriction enzymes were purchased from New England Biolabs (Beijing, China). DNA gel purification kit, and plasmid extraction kit were purchased from TIANGEN BIOTECH (Beijing, China). *Trans*Start® FastPfu DNA Polymerase and T4 DNA ligase were purchased from TransGen Biotech (Beijing, China). ClonExpress MultiS One Step Cloning Kit was purchased from Vazyme (Nanjing, China). Oligonucleotides and genes were synthesized by GENEWIZ (Suzhou, China).

## Plasmid Construction

The plasmids and primers used are listed in Supplementary Table S1 and S2, respectively. The *E. coli*-*C. glutamicum* shuttle vector pXMJ19 (Jakoby et al., 1999) was used for IPTG-inducible expression of the HmuTUV in *C. glutamicum*. The *hmuTUV* gene was amplified by PCR from the genomic DNA of *C. glutamicum* ATCC 13032 with the primer pair *hmuTUV*-F/R. The backbone of pXMJ19 was amplified by PCR with the primer pair pXMJ19-F/R. The DNA fragment was ligated with the linearized pXMJ19, resulting in plasmid pXMJ19-*hmuTUV*.

The IPTG-inducible promoter P*_trc_* of *E. coli*-*C. glutamicum* shuttle vector pEC-XK99E (Kirchner and Tauch, 2003) was substituted to a gluconate-inducible promoter P*_gntK_* for expression of the key genes involved in heme biosynthesis. The DNA fragment of promoter P*_gntK_* was amplified by PCR from the genomic DNA of *C. glutamicum* ATCC 13032 with the primer pair P*_gntK_*-F/R. Then, the fragment was ligated with the linearized pEC-XK99E that was amplified from pEC-XK99E with the primer pair pEC-F/R, resulting in plasmid pEC-P*_gntK_*. The backbone of pEC-P*_gntK_* was amplified by PCR with the primer pair pEC-P*_gntK_*-F/R, and the gene fragments of *pdgS*, *hmbS*, *uroS*, *uroD*, *cgoX*, *cpfC*, and *chdC* were amplified by PCR from the genomic DNA of *C. glutamicum* ATCC 13032 with the primer pairs *pbgS*-F/R, *hmbS*-F/R, *uroS*-F/R, *uroD*-F/R, *cgoX*-F/R, *cpfC*-F/R, or *chdC*-F/R, respectively. The DNA fragments were ligated with the linearized pEC-P*_gntK_* to construct expression plasmids for these genes.

To remove the possible mutations in the plasmid backbone or the chromosome that were randomly introduced during the evolution process, the genes of mutants CpfC^E181G^, CpfC^T194S^, and CpfC^E31V,E100D^ were re-cloned into the original plasmid pEC-P*_gntK_* with the primer pair *cpfC*-F/R. The reconstructed CpfC mutant-overexpressing plasmids and pXMJ19-*hmuTUV* were co-transformed into the original strain *C. glutamicum* ATCC 13032 with a clean background, respectively. The construction procedure of expressing plasmids for mutants PbgS, UroD, and CgoX was same as the above-mentioned process.

The *E. coli* vector pET-21a (Studier et al., 1990) was used for IPTG-inducible expression of the wild-type CpfC and its mutants in strain *E. coli* BL21 (DE3). The *cpfC* gene was amplified by PCR from the genomic DNA of *C. glutamicum* ATCC 13032. The DNA fragment was ligated with the linearized pET-21a that was purified after digestion using the enzymes *Nde* Ⅰ and *Xho* Ⅰ to construct plasmid pET-21a-*cpfC*^WT^. The DNA fragments of CpfC mutants were amplified by PCR from the plasmids pEC-P*_gntK_-cpfC*^E31V,E100D^, pEC-P*_gntK_-cpfC*^E181G^, or pEC-P*_gntK_-cpfC*^T194S^ with the primer pair pET-21a-*cpfC*-F/R, respectively. Then, the DNA fragments were ligated with the linearized pET-21a, resulting in the plasmids pET-21a-*cpfC*^E31V,E100D^, pET-21a-*cpfC*^E181G^, and pET-21a-*cpfC*^T194S^. For the construction of the plasmids pET-21a-*cpfC*^E31V^ and pET-21a-*cpfC*^E100D^, the plasmid pET-21a-*cpfC*^WT^ was linearized with the primer pairs E31V-F/R and E100D-F/R, respectively, and digested with *Dpn* I, phosphorylated with T4 polynucleotide kinase, and self‑ligated using T4 DNA ligase, resulting in the plasmids pET-21a-*cpfC*^E31V^ and pET-21a-*cpfC*^E100D^.

## Protein Expression and Purification

Strain *E. coli* BL21 (DE3) harboring pET-21a-*cpfC*^WT^ and its mutants were cultivated overnight in LB medium at 37℃. Then, 500 μL culture was inoculated to 50 mL LB medium containing 100 μg/mL Amp. After cultivation for 3 h at 37℃ with the OD_600nm_ (optical density at 600 nm) reaching 0.5-0.6, heterologous gene expression was induced by adding 0.5 mM IPTG, and the incubation temperature was shifted to 16℃. Cells were harvested by centrifugation (12,000 × g, 10 min, 4℃) after 24 h of cultivation and the cell pellets were washed and resuspended with Tris-HCl buffer (pH 7.4-7.6). Then, the cell extracts were prepared by sonication. Enzymes were purified using His SpinTrap columns (GE Healthcare, USA) with buffer (20 mM Tris-HCl, 500 mM NaCl, 500 mM imidazole, pH 7.4-7.6), and dialyzed with buffer (20 mM Tris-HCl, 500 mM NaCl, pH 7.4-7.6). The protein concentration was quantified by the Pierce™ BCA Protein Assay Kit (Thermo Fisher Scientific, USA). The purified proteins were analyzed by SDS-PAGE.

## Heme Assay

For seed preparation, strains were cultivated in 24-well plates containing 800 μL TSB medium in each well at 30°C, 800 rpm. After about 12 h of cultivation, the cultivated cells were washed twice with CGXII minimal medium, then inoculated to 800 μL CGXII minimal medium supplemented with 40 g/L glucose, 0.1 mM IPTG, 10 g/L sodium gluconate, 16 μg/mL ZnPPIX, different concentrations of 5-ALA, 5 μg/mL Cm, and 25 μg/mL Km in 24-well plates with an initial OD_600nm_ of 0.1 at 30°C, 800 rpm. After 24 h cultivation, cells were centrifuged (12,000 × g, 10 min, 4℃), and the supernatant was mixed with 0.1 M NaOH at a 1:1 ratio to determine the extracellular heme concentration by the high-performance liquid chromatography (HPLC; 1260 Infinity II, Agilent, Santa Clara, CA) system as previously described (Zhao et al., 2018). For the measurement of intracellular heme, the cell pellets were treated with 1 mL acidic acetone buffer (95:5 ratio of 99% acetone:1.6 M HCl) to extract intracellular heme (Ko et al., 2021), then, the mixture was shaken and mixed. After further centrifugation (12,000 × g, 10 min, 4℃), the supernatants were mixed with 0.1 M NaOH at a 1:1 ratio to determine intracellular heme as above described (Zhao et al., 2018).

# Supplementary Figures and Tables

## Supplementary Figures


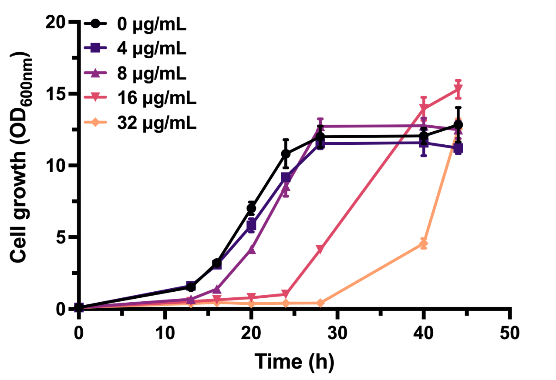


**Supplementary Figure 1.** Effects of different concentrations of ZnPPIX on the cell growth of strain harboring plasmids pXMJ19-*hmuTUV* and pEC-P*_gntK_*. The strains were cultivated in CGXII medium with adding 40 g/L glucose, (0 μg/mL, 4 μg/mL, 8 μg/mL, 16 μg/mL, and 32 μg/mL) ZnPPIX, 10 g/L sodium gluconate, 0.1 mM IPTG, 25 μg/mL Km, and 5 μg/mL Cm. Data are presented as mean values +/− SD (n = 3 independent experiments).


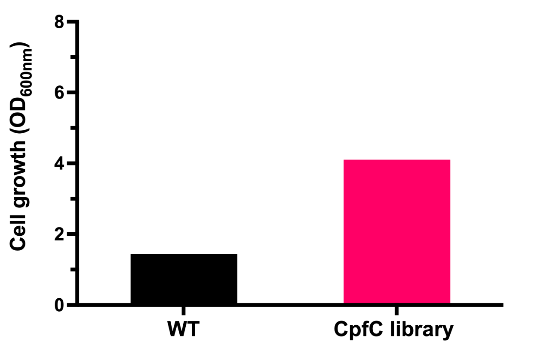


**Supplementary Figure 2.** Cell growth of the strains harboring the CpfC mutation library. The strain harboring plasmids pXMJ19-*hmuTUV* and pEC-P*_gntK_*-*cpfC* was used as the control. These strains were cultivated in CGXII medium with adding 40 g/L glucose, 16 μg/mL ZnPPIX, 10 g/L sodium gluconate, 0.1 mM IPTG, 25 μg/mL Km, and 5 μg/mL Cm for 24 h.


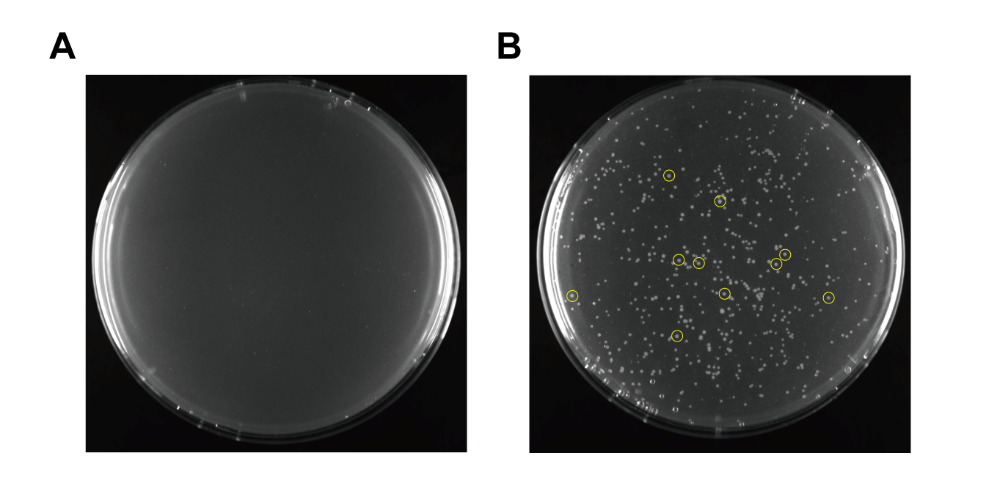


**Supplementary Figure 3.** Cell growth of the strains harboring plasmids pXMJ19-*hmuTUV* and pEC-P*_gntK_*-*cpfC* **(A)** and the CpfC mutant library **(B)** on CGXII agar plate supplemented with 40 g/L glucose, 16 μg/mL ZnPPIX, 10 g/L sodium gluconate, 0.1 mM IPTG, 25 μg/mL Km, and 5 μg/mL Cm. Plates were cultivated for 48 h.


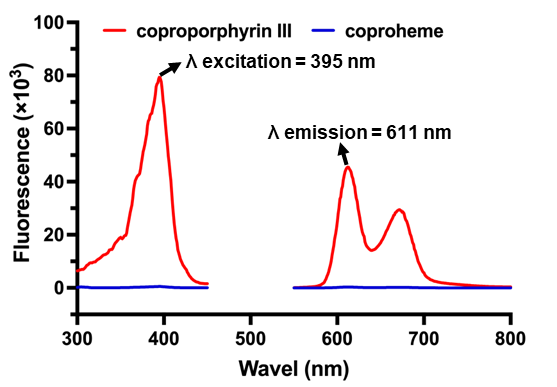


**Supplementary Figure 4.** Spectrofluorimetric scanning of coproporphyrin III and coproheme. For the coproporphyrin III and coproheme standard samples, the excitation wavelength was set from 300 nm to 450 nm, and the emission wavelength was set from 500 nm to 700 nm.


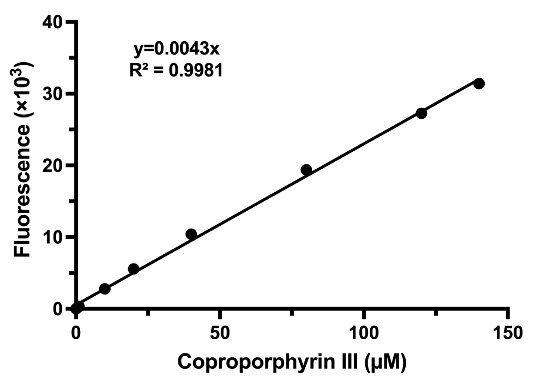


**Supplementary Figure 5.** The linear relationship between the produced fluorescence and different concentration of coproporphyrin III at λ excitation = 395 nm and λ emission = 611 nm.


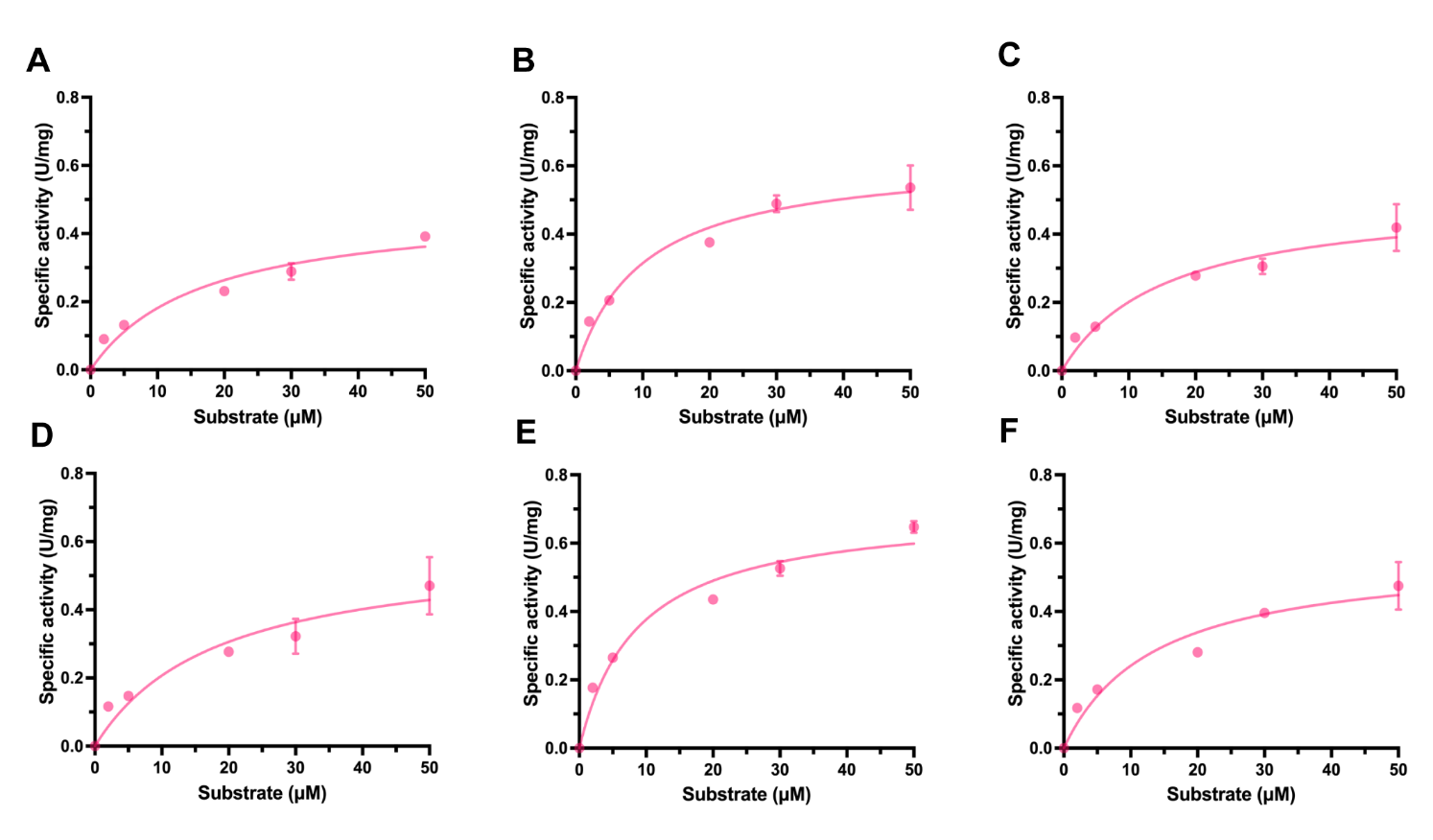


**Supplementary Figure 6.** Michaelis-Menten plots of CpfC^WT^ **(A)**, CpfC^E31V,E100D^ **(B)**, CpfC^E181G^ **(C)**, CpfC^T194S^ **(D)**, CpfC^E31V^ **(E)**, CpfC^E100D^ **(F)**.


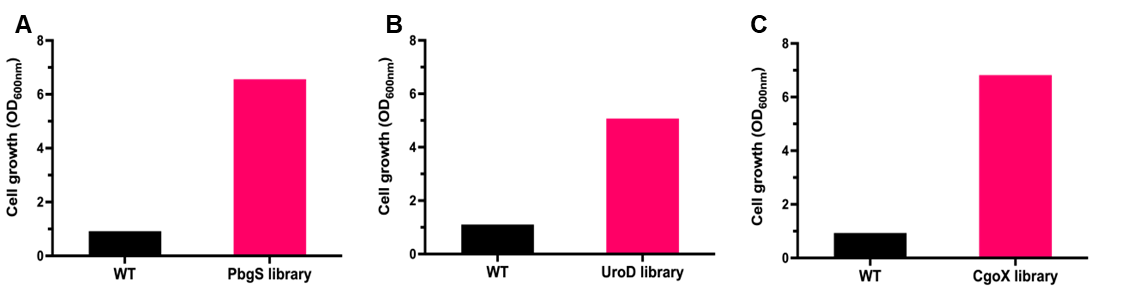


**Supplementary Figure 7.** Cell growth of the strains harboring mutation library of PbgS **(A)**, UroD **(B)**, and CgoX **(C)**. The strains harboring plasmids pXMJ19-*hmuTUV* and pEC-P*_gntK_* overexpressing the wild-type genes were used as the control. These strains were cultivated in CGXII medium with adding 40 g/L glucose, 16 μg/mL ZnPPIX, 10 g/L sodium gluconate, 0.1 mM IPTG, 25 μg/mL Km, and 5 μg/mL Cm for 24 h.

## Supplementary Tables

**Supplementary Table 1.** Bacterial strains and plasmids used in this study.

| **Strain or Plasmid** | **Relevant characteristic** | **Source** |
| --- | --- | --- |
| **Strains** |  |  |
| *E. coli* *Trans*1 T1 | Host for cloning | TransGen Biotech |
| *E. coli* BL21 (DE3) | Protein expression and purification | TransGen Biotech |
| *C. glutamicum*  ATCC 13032 | Wild-type strain | ATCC |
| ATCC 13032  (pXMJ19-*hmuTUV*) | Derivative of strain ATCC 13032 harboring pXMJ19-*hmuTUV* | This study |
| ATCC 13032  (pXMJ19-*hmuTUV*  and pEC-P*_gntK_-pbgS*) | Derivative of strain ATCC 13032 harboring pXMJ19-*hmuTUV* and pEC-P*_gntK_-pbgS* | This study |
| ATCC 13032  (pXMJ19-*hmuTUV*  and pEC-P*_gntK_-hmbS*) | Derivative of strain ATCC 13032 harboring pXMJ19-*hmuTUV* and pEC-P*_gntK_-hmbS* | This study |
| ATCC 13032  (pXMJ19-*hmuTUV*  and pEC-P*_gntK_-uroS*) | Derivative of strain ATCC 13032 harboring pXMJ19-*hmuTUV* and pEC-P*_gntK_-uroS* | This study |
| ATCC 13032  (pXMJ19-*hmuTUV*  and pEC-P*_gntK_-uroD*) | Derivative of strain ATCC 13032 harboring pXMJ19-*hmuTUV* and pEC-P*_gntK_-uroD* | This study |
| ATCC 13032  (pXMJ19-*hmuTUV*  and pEC-P*_gntK_-cgoX*) | Derivative of strain ATCC 13032 harboring pXMJ19-*hmuTUV* and pEC-P*_gntK_-cgoX* | This study |
| ATCC 13032  (pXMJ19-*hmuTUV*  and pEC-P*_gntK_-cpfC*) | Derivative of strain ATCC 13032 harboring pXMJ19-*hmuTUV* and pEC-P*_gntK_-cpfC* | This study |
| ATCC 13032  (pXMJ19-*hmuTUV*  and pEC-P*_gntK_-chdC*) | Derivative of strain ATCC 13032 harboring pXMJ19-*hmuTUV* and pEC-P*_gntK_-chdC* | This study |
| ATCC 13032  (pXMJ19-*hmuTUV* and  pEC-P*_gntK_*-*cpfC*^E31V,E100D^) | Derivative of strain ATCC 13032 harboring pXMJ19-*hmuTUV* and pEC-P*_gntK_-cpfC*^E31V,E100D^ | This study |
| ATCC 13032  (pXMJ19-*hmuTUV* and  pEC-P*_gntK_*-*cpfC*^E181G^) | Derivative of strain ATCC 13032 harboring pXMJ19-*hmuTUV* and pEC-P*_gntK_-cpfC*^E181G^ | This study |
| ATCC 13032  (pXMJ19-*hmuTUV* and  pEC-P*_gntK_*-*cpfC*^T194S^) | Derivative of strain ATCC 13032 harboring pXMJ19-*hmuTUV* and pEC-P*_gntK_-cpfC*^T194S^ | This study |
| ATCC 13032  (pXMJ19-*hmuTUV* and  pEC-P*_gntK_-pbgS*1) | Derivative of strain ATCC 13032 harboring pXMJ19-*hmuTUV* and pEC-P*_gntK_-pbgS*1 | This study |
| ATCC 13032  (pXMJ19-*hmuTUV* and  pEC-P*_gntK_-pbgS*2) | Derivative of strain ATCC 13032 harboring pXMJ19-*hmuTUV* and pEC-P*_gntK_-pbgS*2 | This study |
| ATCC 13032  (pXMJ19-*hmuTUV* and  pEC-P*_gntK_-pbgS*3) | Derivative of strain ATCC 13032 harboring pXMJ19-*hmuTUV* and pEC-P*_gntK_-pbgS*3 | This study |
| ATCC 13032  (pXMJ19-*hmuTUV* and  pEC-P*_gntK_-pbgS*4） | Derivative of strain ATCC 13032 harboring pXMJ19-*hmuTUV* and pEC-P*_gntK_-pbgS*4 | This study |
| ATCC 13032  (pXMJ19-*hmuTUV* and  pEC-P*_gntK_-pbgS*5) | Derivative of strain ATCC 13032 harboring pXMJ19-*hmuTUV* and pEC-P*_gntK_-pbgS*5 | This study |
| ATCC 13032  (pXMJ19-*hmuTUV* and  pEC-P*_gntK_-uroD*1) | Derivative of strain ATCC 13032 harboring pXMJ19-*hmuTUV* and pEC-P*_gntK_-uroD*1 | This study |
| ATCC 13032  (pXMJ19-*hmuTUV* and  pEC-P*_gntK_-uroD*2) | Derivative of strain ATCC 13032 harboring pXMJ19-*hmuTUV* and pEC-P*_gntK_-uroD*2 | This study |
| ATCC 13032  (pXMJ19-*hmuTUV* and  pEC-P*_gntK_-uroD*3) | Derivative of strain ATCC 13032 harboring pXMJ19-*hmuTUV* and pEC-P*_gntK_-uroD*3 | This study |
| ATCC 13032  (pXMJ19-*hmuTUV* and  pEC-P*_gntK_-cgoX*1) | Derivative of strain ATCC 13032 harboring pXMJ19-*hmuTUV* and pEC-P*_gntK_-cgoX*1 | This study |
| ATCC 13032  (pXMJ19-*hmuTUV* and  pEC-P*_gntK_-cgoX*2) | Derivative of strain ATCC 13032 harboring pXMJ19-*hmuTUV* and pEC-P*_gntK_-cgoX*2 | This study |
| ATCC 13032  (pXMJ19-*hmuTUV* and  pEC-P*_gntK_-cgoX*3) | Derivative of strain ATCC 13032 harboring pXMJ19-*hmuTUV* and pEC-P*_gntK_-cgoX*3 | This study |
| ATCC 13032  (pXMJ19-*hmuTUV* and  pEC-P*_gntK_-cgoX*4) | Derivative of strain ATCC 13032 harboring pXMJ19-*hmuTUV* and pEC-P*_gntK_-cgoX*4 | This study |
| BL21 (pET-21a-*cpfC*) | Derivative of strain *E. coli* BL21 (DE3) harboring pET-21a-*cpfC* | This study |
| BL21 (pET-21a-*cpfC*^E31V,E100D^) | Derivative of strain *E. coli* BL21 (DE3) harboring pET-21a-*cpfC*^E31V,E100D^ | This study |
| BL21 (pET-21a-*cpfC*^E31V^) | Derivative of strain *E. coli* BL21 (DE3) harboring pET-21a-*cpfC*^E31V^ | This study |
| BL21 (pET-21a-*cpfC*^E100D^) | Derivative of strain *E. coli* BL21 (DE3) harboring pET-21a-*cpfC*^E100D^ | This study |
| BL21 (pET-21a-cpfC^E181G^) | Derivative of strain *E. coli* BL21 (DE3) harboring pET-21a-*cpfC*^E181G^ | This study |
| BL21 (pET-21a-cpfC^T194S^) | Derivative of strain *E. coli* BL21 (DE3) harboring pET-21a-*cpfC*^T194S^ | This study |
| **Plasmids** |  |  |
| pXMJ19 | Expression vector of *C. glutamicum*, IPTG-inducible promoter P*_tac_*, Cm^R^ | (Jakoby et al., 1999) |
| pEC-XK99E | Expression vector of *C. glutamicum*, IPTG-inducible promoter P*_trc_*, Km^R^ | (Kirchner and Tauch, 2003) |
| pET-21a | ColE1, Amp^R^, *lacI*, T7 promoter, C-His | Lab stock |
| pXMJ19-*hmuTUV* | pXMJ19 derivative carrying the *hmuTUV* | This study |
| pEC-P*_gntK_* | Expression vector of *C. glutamicum*, gluconate-inducible promoter P*_gntK_*, Km^R^ | This study |
| pEC-P*_gntK_*-*pdgS* | pEC-P*_gntK_* derivative carrying the *pdgS* | This study |
| pEC-P*_gntK_*-*hmbS* | pEC-P*_gntK_* derivative carrying the *hmbS* | This study |
| pEC-P*_gntK_*-*uroS* | pEC-P*_gntK_* derivative carrying the *uroS* | This study |
| pEC-P*_gntK_*-*uroD* | pEC-P*_gntK_* derivative carrying the *uroD* | This study |
| pEC-P*_gntK_*-*cgoX* | pEC-P*_gntK_* derivative carrying the c*goX* | This study |
| pEC-P*_gntK_*-*cpfC* | pEC-P*_gntK_* derivative carrying the *cpfC* | This study |
| pEC-P*_gntK_*-*chdC* | pEC-P*_gntK_* derivative carrying the *chdC* | This study |
| pEC-P*_gntK_-cpfC*^E31V,E100D^ | pEC-P*_gntK_* derivative carrying the *cpfC*^E31V,E100D^ mutant | This study |
| pEC-P*_gntK_*-*cpfC*^T194S^ | pEC-P*_gntK_* derivative carrying the c*pfC*^T194S^ mutant | This study |
| pEC-P*_gntK_*-*cpfC*^E181G^ | pEC-P*_gntK_* derivative carrying the c*pfC*^E181G^ mutant | This study |
| pEC-P*_gntK_*-*pbgS*1 | pEC-P*_gntK_* derivative harboring *pbgS*1 | This study |
| pEC-P*_gntK_*-*pbgS*2 | pEC-P*_gntK_* derivative harboring *pbgS*2 | This study |
| pEC-P*_gntK_*-*pbgS*3 | pEC-P*_gntK_* derivative harboring *pbgS*3 | This study |
| pEC-P*_gntK_*-*pbgS*4 | pEC-P*_gntK_* derivative harboring *pbgS*4 | This study |
| pEC-P*_gntK_*-*pbgS*5 | pEC-P*_gntK_* derivative harboring *pbgS*5 | This study |
| pEC-P*_gntK_*-*uroD*1 | pEC-P*_gntK_* derivative harboring *uroD*1 | This study |
| pEC-P*_gntK_*-*uroD*2 | pEC-P*_gntK_* derivative harboring *uroD*2 | This study |
| pEC-P*_gntK_*-*uroD*3 | pEC-P*_gntK_* derivative harboring *uroD*3 | This study |
| pEC-P*_gntK_*-*cgoX*1 | pEC-P*_gntK_* derivative harboring *cgoX*1 | This study |
| pEC-P*_gntK_*-*cgoX*2 | pEC-P*_gntK_* derivative harboring *cgoX*2 | This study |
| pEC-P*_gntK_*-*cgoX*3 | pEC-P*_gntK_* derivative harboring *cgoX*3 | This study |
| pEC-P*_gntK_*-*cgoX*4 | pEC-P*_gntK_* derivative harboring *cgoX*4 | This study |
| pET-21a-*cpfC*^WT^ | pET-21a derivative with *cpfC*, C-terminal 6-His tag | This study |
| pET-21a-*cpfC*^E31V,E100D^ | pET-21a derivative with *cpfC*^E31V,E100D^ mutant, C-terminal 6-His tag | This study |
| pET-21a-*cpfC*^T194S^ | pET-21a derivative with *cpfC*^T194S^ mutant, C-terminal 6-His tag | This study |
| pET-21a-*cpfC*^E181G^ | pET-21a derivative with *cpfC*^E181G^ mutant, C-terminal 6-His tag | This study |
| pET-21a-*cpfC*^E31V^ | pET-21a derivative with *cpfC*^E31V^ mutant, C-terminal 6-His tag | This study |
| pET-21a-*cpfC*^E100D^ | pET-21a derivative with *cpfC*^E100D^ mutant, C-terminal 6-His tag | This study |

Km^R^, Cm^R^ and Amp^R^ represent resistance to kanamycin, chloramphenicol, and ampicillin, respectively.

**Supplementary Table 2.** Primers used in this study.

| **Primer** | **Sequence (5’-3’)** | **Relevance** |
| --- | --- | --- |
| pXMJ19-F | AGCTTGGCTGTTTTGGCG | PCR amplification of fragment for pXMJ19-*hmuTUV* construction |
| pXMJ19-R | GAAATTGTTATCCGCTCACA |  |
| *hmuTUV*-F | TGTGAGCGGATAACAATTTCGCAACCCGACTGGAAAATAAG |  |
| *hmuTUV*-R | CGCCAAAACAGCCAAGCTCTAAGCTGGTGAAACCGTATTTTTC |  |
| pEC-F | ACTTTAAGAAGGAGATATCATGATCCTCTAGAGTCGACCTGCA | PCR amplification of fragment for pEC-P*_gntK_* |
| pEC-R | TGAAATACCGCACAGATGCGTA |  |
| P*_gntK_*-F | TACGCATCTGTGCGGTATTTCAGTATCAATGGAATCCGGGACG |  |
| P*_gntK_*-R | ATGATATCTCCTTCTTAAAGTTCAGACAATATGTAAGCCTTCGG |  |
| pEC-P*_gntK_*-R | CCGAAGGCTTACATATTGTCTGAACTTTAAGAAGGAGATA | PCR amplification of pEC*-*P*_gntK_* fragment for genes overexpression plasmid construction |
| pEC-P*_gntK_*-F | GATCCTCTAGAGTCGACCTGCAG |  |
| *pbgS*-F | TTAAGAAGGAGATATCATATGAGCACTTCTTCTGATTACTCCC | PCR amplification of fragment for pEC-P*_gntK-_pdgS* |
| *pbgS*-R | GGTCGACTCTAGAGGATCTTAAGCGTTTCGCAGTGCG |  |
| *hmbS*-F | TTAAGAAGGAGATATCATATGACCTTAAAAATTGGTACCCGAG | PCR amplification of fragment for pEC-P*_gntK-_hmbS* |
| *hmbS*-R | GGTCGACTCTAGAGGATCTTAGCTGCGGTCGCCGAG |  |
| *uroS*-F | TTAAGAAGGAGATATCATATGACTATCGCCCATAAGCCC | PCR amplification of fragment for pEC-P*_gntK_-uroS* |
| *uroS*-R | GGTCGACTCTAGAGGATCTTAAGACGCTTTTCGACGCCT |  |
| *uroD*-F | TTAAGAAGGAGATATCATATGTCTGCTCTTACTATTCCAGCTG | PCR amplification of fragment for pEC-P*_gntK_-uroD* |
| *uroD*-R | GGTCGACTCTAGAGGATCTTAAGAATGAATGATGGAGACGG |  |
| *cgoX*-F | TTAAGAAGGAGATATCATGTGCGTTTTGCCATCATCGG | PCR amplification of fragment for pEC-P*_gntK_-cgoX* |
| *cgoX*-R | GGTCGACTCTAGAGGATCTTATCCCAGCAACCTGTGTACTGC |  |
| *cpfC*-F | TTAAGAAGGAGATATCATATGAATGAACGCACATCGGAT | PCR amplification of fragment for pEC-P*_gntK_-cpfC* |
| *cpfC*-R | GGTCGACTCTAGAGGATCCTAGTTGGCAGCTGGCGC |  |
| *chdC*-F | TTAAGAAGGAGATATCATGTGAGCGAGCTCGATATTAAAC | PCR amplification of fragment for pEC-P*_gntK_-chdC* |
| *chdC*-R | GGTCGACTCTAGAGGATCTTAAGGAAGAACCTTAATCAGATCT |  |
| pEC-P*_gntK_*-1 | GATCCTCTAGAGTCGACCTGCAG | PCR amplification of pEC-P*_gntK_* fragment for mutation library construction |
| pEC-P*_gntK_*-2 | ATGATATCTCCTTCTTAAAGTTCAGACAATATGTAAGCCTTCGG |  |
| *pbgS*-1 | CTTTAAGAAGGAGATATCATATGAGCACTTCTTCTGATTACTCCC | Error-PCR amplification of fragment for p*bgS* mutation library construction |
| *pbgS*-2 | CAGGTCGACTCTAGAGGATCTTAAGCGTTTCGCAGTGCG |  |
| *uroD*-1 | CTTTAAGAAGGAGATATCATATGTCTGCTCTTACTATTCCAGCTG | Error-PCR amplification of fragment for *uroD* mutation library construction |
| *uroD*-2 | CAGGTCGACTCTAGAGGATCTTAAGAATGAATGATGGAGACGG |  |
| *cgoX*-1 | CTTTAAGAAGGAGATATCATGTGCGTTTTGCCATCATCG | Error-PCR amplification of fragment for *cgoX* mutation library construction |
| *cgoX*-2 | CAGGTCGACTCTAGAGGATCTTATCCCAGCAACCTGTGTACTG |  |
| *cpfC*-1 | CTTTAAGAAGGAGATATCGATCCTCTAGAGTCGACCTGCAG | Error-PCR amplification of fragment for *cpfC* mutation library construction |
| *cpfC*-2 | CAGGTCGACTCTAGAGGATCCTAGTTGGCAGCTGGCGC |  |
| pET-21a-*cpfC*-F | TAAGAAGGAGATATACATATGAATGAACGCACATCG | PCR amplification of fragment for pET-21a-*cpfC*^WT^ and its mutant |
| pET-21a-*cpfC*-R | GTGGTGGTGGTGCTCGAGGTT GGCAGCTGGCGCC |  |
| E31V-F | TTTGGTGAATGTCACTCACGG AAGGG | PCR amplification of fragment for pET-21a-*cpfC*^E31V^ |
| E31V-R | AACGGACGAACCTCCTCGT |  |
| E100D-F | GCTGATCAAATGGCTGATGAC GGC | PCR amplification of fragment for pET-21a-*cpfC*^E100D^ |
| E100D-R | GGCCTCATTATCAAACGGCT |  |

**Supplementary Table 3.** The amino acid substitutions in mutants screened from growth-coupled selection system.

| **Mutant** | **Amino acid change** |
| --- | --- |
| CpfC^E31V,E100D^ | Gly31Val, Glu100Asp |
| CpfC^E181G^ | Glu181Gly |
| CpfC^T194S^ | Thr194Ser |
| PbgS1 | Met42Thr, Ala192Ser |
| PbgS2 | Asp132Gly, Ser244Pro |
| PbgS3 | Ile204Thr, Ala206Thr, Ala212Thr, Leu265Pro, Ala283Val, Ala297Thr, Tyr397His, Ala327Ser |
| PbgS4 | Asp132Gly, Ser244Pro |
| PbgS5 | Arg13His, Pro41Ser, Asp304Gly |
| UroD1 | Ala79Val, Leu120Pro, Gly337Asp |
| UroD2 | Ala320Thr |
| UroD3 | Ile19Thr, Pro71Val |
| CgoX1 | Gln195Leu, Val343Met |
| CgoX2 | Gly245Asp, Ala269Thr, Val364Ala |
| CgoX3 | Lys200Glu, Thr211Ala, Ala229Ala, Lys242Asn, Gly255Ser, Ser408Pro |
| CgoX4 | Pro45Ser, Ala99Pro, Ala354Thr |

# References

Jakoby, M., Ngouoto-Nkili, C.-E., and Burkovski, A. (1999). Construction and application of new *Corynebacterium glutamicum* vectors. *Biotechnol. Tech.* 13 (6), 437-441. doi: 10.1023/A:1008968419217

Kirchner, O., and Tauch, A. (2003). Tools for genetic engineering in the amino acid-producing bacterium *Corynebacterium glutamicum*. *J. Biotechnol.* 104 (1-3), 287-299. doi: 10.1016/s0168-1656(03)00148-2

Ko, Y.J., Kim, M., You, S.K., Shin, S.K., Chang, J., Choi, H.J., et al. (2021). Animal-free heme production for artificial meat in *Corynebacterium glutamicum* via systems metabolic and membrane engineering. *Metab. Eng.* 66, 217-228. doi: 10.1016/j.ymben.2021.04.013

Studier, F.W., Rosenberg, A.H., Dunn, J.J., and Dubendorff, J.W. (1990). Use of T7 RNA polymerase to direct expression of cloned genes. *Methods Enzymol.* 185, 60-89. doi: 10.1016/0076-6879(90)85008-c

Zhao, X.R., Choi, K.R., and Lee, S.Y. (2018). Metabolic engineering of *Escherichia coli* for secretory production of free haem. *Nat. Catal.* 1 (9), 720-728. doi: 10.1038/s41929-018-0126-1
